# *Bcl-2* is a critical mediator of intestinal transformation

**DOI:** 10.1038/ncomms10916

**Published:** 2016-03-09

**Authors:** Maartje van der Heijden, Cheryl D. Zimberlin, Anna M. Nicholson, Selcuk Colak, Richard Kemp, Sybren L. Meijer, Jan Paul Medema, Florian R. Greten, Marnix Jansen, Douglas J. Winton, Louis Vermeulen

**Affiliations:** 1 Cancer Research UK, Cambridge Institute, University of Cambridge, Robinson Way, Cambridge CB2 0RE, UK; 2 Laboratory for Experimental Oncology and Radiobiology (LEXOR), Center for Experimental Molecular Medicine (CEMM), Academic Medical Center, Meibergdreef 9, 1105 AZ Amsterdam, The Netherlands; 3 Department of Pathology, Academic Medical Center, Meibergdreef 9, 1105 AZ Amsterdam, The Netherlands; 4 Cancer Genomics Center, Center for Molecular Medicine, HP Stratenum 3.217, Universiteitsweg 100, 3584 CG Utrecht, The Netherlands; 5 Institute for Tumor Biology and Experimental Therapy, Georg-Speyer-Haus, Paul-Ehrlich-Straße 42-44, 60596 Frankfurt, Germany; 6 Barts Cancer Institute, Barts and the London School of Medicine and Dentistry, Queen Mary University of London, London EC1M 6BQ, UK

## Abstract

Intestinal tumour formation is generally thought to occur following mutational events in the stem cell pool. However, active NF-κB signalling additionally facilitates malignant transformation of differentiated cells. We hypothesized that genes shared between NF-κB and intestinal stem cell (ISCs) signatures might identify common pathways that are required for malignant growth. Here, we find that the NF-κB target *Bcl-2*, an anti-apoptotic gene, is specifically expressed in ISCs in both mice and humans. *Bcl-2* is dispensable in homeostasis and, although involved in protecting ISCs from radiation-induced damage, it is non-essential in tissue regeneration. *Bcl-2* is upregulated in adenomas, and its loss or inhibition impairs outgrowth of oncogenic clones, because *Bcl-2* alleviates apoptotic priming in epithelial cells following *Apc* loss. Furthermore, *Bcl-2* expression in differentiated epithelial cells renders these cells amenable to clonogenic outgrowth. Collectively, our results indicate that *Bcl-2* is required for efficient intestinal transformation following *Apc*-loss and constitutes a potential chemoprevention target.

## Abstract (web summary)

The anti-apoptotic protein Bcl-2 is selectively expressed in intestinal stem cells (ISCs). Here, the authors show that, in intestinal stem cells, Bcl-2 alleviates apoptotic priming induced by the loss of the tumour suppressor Apc in ISCs and that the absence of Bcl-2 or pharmacological blockade of Bcl-2 can inhibit the intestinal tumorigenesis driven by the Apc-loss.

## Graphical abstract

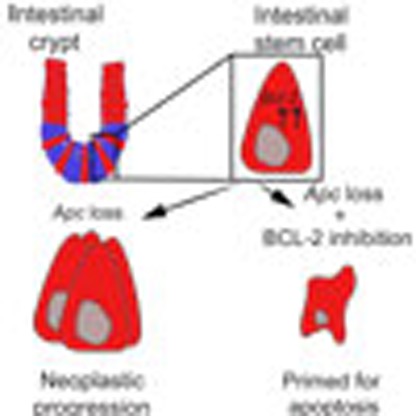

Colorectal cancer (CRC) is the third most common cancer worldwide, with ∼1.2 million new diagnoses and over 600,000 fatalities each year^1^. Adequate treatment is challenging and the development of new preventive and therapeutic strategies remains crucial. CRC formation is a stepwise process during which various histopathological stages can be detected^2^. Each stage is characterized by an accumulation of genetic aberrations that provide a competitive advantage to clones that arise following their occurrence^3^,^4^. Previously it has been reported that transformation occurs most efficiently following mutation in the intestinal stem cell (ISC) compartment^5^. For example, activation of the Wnt pathway, commonly arising following inactivation of the *Apc* gene, specifically in ISCs rapidly results in tumour formation, whereas, *Apc* inactivation in more differentiated cells fails to induce tumour growth^5^,^6^,^7^,^8^. This suggests that ISCs are the cell of origin of a large proportion of CRCs. Critically, the molecular mechanism by which ISCs more readily generate pre-neoplastic expansions on acquisition of mutations than their differentiated descendants is unknown. We hypothesized that identifying the mediators of ISC susceptibility to transformation might suggest potentially attractive chemopreventive and therapeutic targets.

Clues to identify signals associated with CRC development might be derived from conditions that affect the risk of onset of the disease. For example, inflammatory bowel disease is associated with an increased susceptibility to develop CRC^9^. Reciprocally, usage of compounds with anti-inflammatory properties such as non-steroidal anti-inflammatory drugs, including celecoxib and aspirin, are associated with a small, but significant reduction in respectively colorectal adenoma and carcinoma incidence and likely impact on tumour initiation^10^,^11^,^12^. Moreover, intestinal inflammation has recently been shown to increase the pool of cells susceptible to oncogenic transformation to include intestinal differentiated cells (IDC)^7^,^8^. Specifically, activation of the NF-κB pathway in differentiated cells, which is an important mediator of the intestinal inflammatory response, renders these cells capable of generating tumours on inactivation of the *Apc* gene^7^. Conversely, genetic inactivation of the NF-κB pathway, either directly by *p65*-inactivation or by reducing reactive oxygen species following *Rac1*-loss, resulted in a reduced tumour incidence, further suggesting a critical role for this pathway in CRC genesis^13^,^14^.

Here we report that ISCs exhibit an intrinsic activation of the NF-κB pathway. Moreover, we show that B-cell lymphoma 2 (*Bcl-2)*, a key NF-κB target gene, is highly expressed in ISCs. Genetic as well as pharmacological inactivation of BCL-2 impairs adenoma formation and results in formation of indolent lesions similar to cyst-like structures observed when oncogenic mutations are introduced in the differentiated cell pool. Furthermore, *Bcl-2* overexpression in differentiated cells generates clonogenic cells on *Apc*-loss. Our data thus reveals that *Bcl-2* is an important mediator of transformation of intestinal epithelial cells but its loss bears no impact on the intestine in homeostasis. Therefore future studies are warranted to explore if intestine-specific BCL-2 inhibition could be used as a chemopreventive strategy for CRC.

## Results

### ISCs display high NF-κB activity and target gene expression

**Figure 1:**
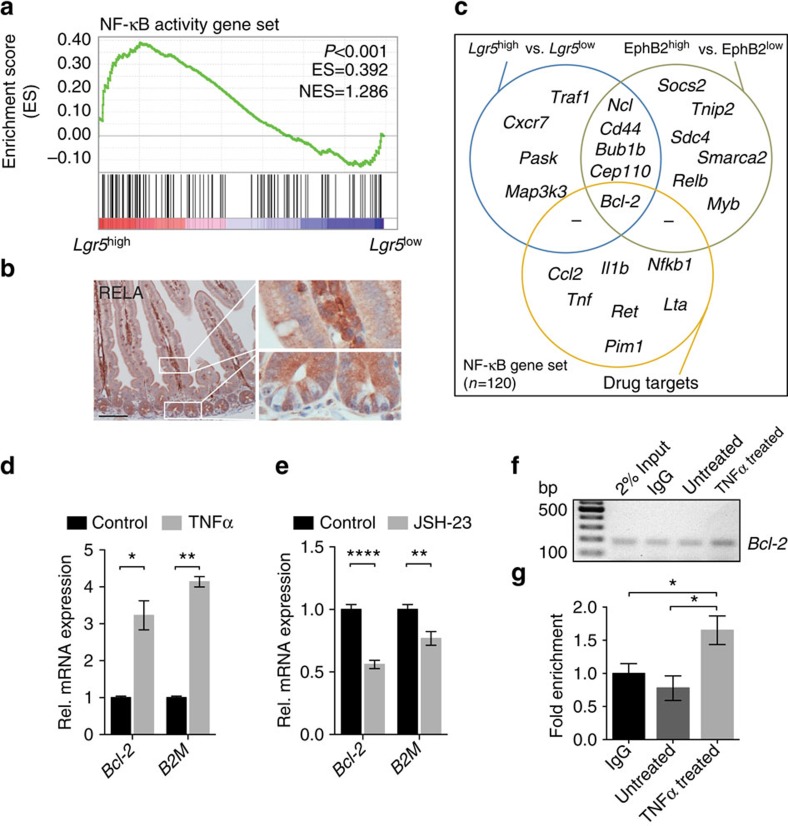
ISCs exhibit high NF-κB pathway activity. (**a**) GSEA demonstrates higher NF-κB target gene expression in *Lgr5*-GFP^high^ versus *Lgr5*-GFP^low^ cells. ES, enrichment score; NES, normalized enrichment score. The NF-κB signature was adapted from Compagno *et al.* (*n*=120 genes)^48^. (**b**) Immunohistochemistry for RELA in murine small intestinal tissue. Nuclear staining of RELA indicates a high activity of the NF-κB pathway in the ISC compartment compared with the differentiated cell lineages. Scale bar, 100 μm. (**c**) Venn diagram indicating the significantly (*P*<0.01) differentially expressed genes for the indicated conditions (left and right circle) and genes listed in the gene category ‘drug targets' (bottom circle) of the NF-κB pathway within the NF-κB target gene signature. (**a**,**c**) Gene expression profiles of *Lgr5*-GFP^high/low^ and EphB2^high/low^ are obtained from GSE33948 and GSE27605, respectively. (**d**) Treatment of intestinal organoids with TNFα for 24 h increases *Bcl-2* and *B2M* expression. The latter serving as a positive control for NF-kB activation. Error bars represent the s.e.m. (*n*=2 independent experiments), *P<0.05, **P<0.01, Student's *t*-test. (**e**) Treatment of intestinal organoids with the NF-κB inhibitor JSH-23 for 24 h decreases *Bcl-2* and *B2M* expression. Error bars represent the s.e.m. (*n*=4 independent experiments), **P<0.01, ****P<0.0001, Student's *t*-test. (**f**) ChIP assay with a p65 antibody on DNA isolated from untreated and TNFα treated organoids. PCR showing enrichment for the *Bcl-2* promoter region in the TNFα treated group. (**g**) Quantitative PCR analysis on DNA derived from the ChIP assay and using the same primer set to detect the *Bcl-2* promoter region as shown in **f**. Error bars indicate the s.e.m. (*n*=2 independent ChIP assays), *P<0.05, Student's *t*-test.

Intestinal epithelium transformation occurs most efficiently in the ISC compartment^5^. As NF-κB signalling has been shown to be a critical component for transformation to occur, we investigated whether NF-κB signalling activity was increased in the ISC compartment^7^,^8^. Gene set enrichment analysis (GSEA) revealed an increased expression of NF-κB target genes in *Lgr5*^high^ versus *Lgr5*^low^ cells (Fig. 1a). Moreover, when compared with neighbouring Paneth cells and other differentiated cells, increased NF-κB pathway activity was observed in the crypt base, where the ISCs reside, as evidenced by nuclear accumulation of RELA/p65 (Fig. 1b). To identify additional genes required for the transformation of NF-κB^low^ IDCs but not NF-κB^high^ ISCs, we identified NF-κB target genes that are most differentially expressed between ISCs (*Lgr5*-GFP^high^, EphB2^high^) and IDCs (*Lgr5*-GFP^low^, EphB2^low^). This revealed five genes: *Ncl*, *Cd44*, *Cep110*, *Bub1b* and *Bcl-2*, to be highly significantly (*P*<0.01) differentially expressed in ISCs versus IDCs in various public data sets (Fig. 1c; Supplementary Fig. 1)^15^,^16^. Because *Bcl-2* is a gene that is well known for its anti-apoptotic properties and the only gene we identified that can be effectively pharmacologically inhibited, we focused our attention on this candidate. To confirm that *Bcl-2* is an NF-κB target gene in intestinal epithelial cells we treated organoid cultures with TNFα to enhance NF-κB activity, as well as with an NF-κB inhibitor (JSH-23), resulting in increased and decreased *Bcl-2* messenger RNA levels respectively (Fig. 1d,e). Furthermore, we observed an increase in p65 binding to the *Bcl-2* promoter on TNFα stimulation of intestinal epithelial cells (Fig. 1f,g; Supplementary Fig. 10). Thus, *Bcl-2* is a direct NF-κB target within the intestinal epithelium.

### BCL-2 marks crypt base columnar stem cells in the intestine

**Figure 2:**
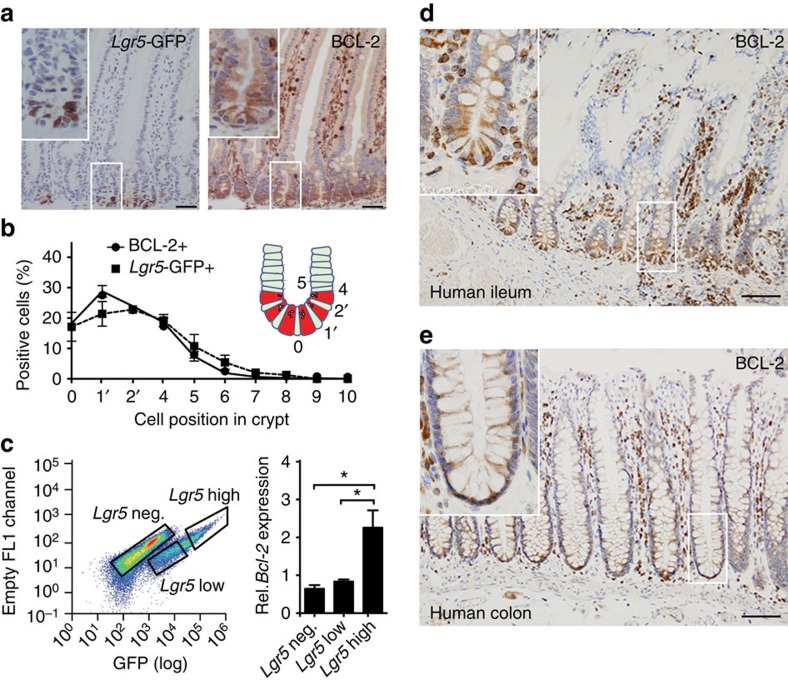
***Bcl-2*** **marks crypt base columnar ISCs.** (**a**) Immunohistochemistry for GFP (left) and BCL-2 (right) stained sections of the small intestine of *Lgr5*-GFP mice. BCL-2 staining shows a similar expression distribution as the *Lgr5*-GFP^+^ cells within crypts. Scale bars, 50 μm. (**b**) Graph depicting the percentage of cells at particular cell positions within crypts that are positive for *Lgr5*-GFP and BCL-2. A similar distribution of crypt base specific expression can be found in both cases. Error bars represent s.e.m. (*n*=3 mice). (**c**) FACS graph indicating the sorting gates for *Lgr5*-GFP^negative^, *Lgr5*-GFP^low^ and *Lgr5*-GFP^high^ cell populations (left) and bar graph depicting the relative messenger RNA (mRNA) expression of *Bcl-2* within the indicated populations showing the highest expression in the *Lgr5*-GFP^high^ population (right panel). Error bars represent the s.e.m. (*n*=3 mice), *P<0.05, Student's *t*-test. (**d**) BCL-2 staining of human ileum tissue. Scale bar, 50 μm. (**e**) BCL-2 staining of human colonic tissue. Scale bar, 50 μm.

Previously immunohistochemical analyses revealed that BCL-2 is mostly expressed within the base of both human and murine intestinal crypts^17^,^18^. Indeed we found that high BCL-2 expressing cells correlates to the *Lgr5*-green fluorescent protein (GFP)+ crypt base columnar cell population (Fig. 2a,b). Fluorescence-activated cell sorting (FACS) of various *Lgr5*-GFP expressing populations (negative, low and high) confirms this observation and reveals that *Lgr5*-GFP^neg.^ and *Lgr5*-GFP^low^ cells, representing differentiated and transit-amplifying cells respectively, display comparable low levels of *Bcl-2* expression (Fig. 2c). High *Bcl-2* transcript levels are detected only in the *Lgr5*-GFP^high^ ISCs. These results were supported by immunohistochemistry for BCL-2 on human intestinal tissue (Fig. 2d,e). In the distal small intestine (ileum) BCL-2 expression was mostly restricted to the crypt base columnar cells (Fig. 2d). Higher expression was also seen in the crypt base of human colon albeit less restricted to a specific population in line with the more indistinct nature of *Lgr5*^+^ ISCs in this tissue (Fig. 2e).

### *Bcl-2* is dispensable for intestinal homeostasis

To study the functional relevance of *Bcl-2* in the intestine we generated *Bcl-2* knockout mice by crossing a previously described Cre-inducible *Bcl-2* knockout strain (*Bcl-2*^fl/fl^)^19^, which allows for inactivation of the gene by removing the second exon, with a germ-line *Cre* deleter strain (*PGK*-Cre; Supplementary Fig. 2a,b). These *Bcl-2*^−/−^ mice displayed characteristic features that have been reported before in the context of *Bcl-2* knockout animals: they are of reduced size compared with both *Bcl-2*^+/+^ and *Bcl-2*^+/−^ littermates and display an accelerated greying of the fur (Supplementary Fig. 3a)^20^,^21^,^22^. Following confirmation of the lack of *Bcl-2* expression both in the intestinal epithelial cell and the lymphocytic cell compartment (Supplementary Fig. 3b), we evaluated intestinal tissue morphology and differentiation patterns (Supplementary Fig. 3c–f). Alcian blue, lysozyme and villin staining did not reveal alterations in the sizes or distributions of the goblet cell, Paneth cell or total enterocyte populations, respectively (Supplementary Fig. 3d). Furthermore, *Bcl-2* loss bears no impact on the amount or distribution of proliferating cells in the intestine (Supplementary Fig. 3e) and a small apparent reduction in the number of apoptotic cells was not found to be significant (Supplementary Fig. 3f). These results show that no cellular and intestinal tissue phenotype could be detected in *Bcl-2* deficient mice, and therefore suggest that this gene is dispensable for normal intestinal homeostasis. To what degree the reduced body size and weight is due to impaired intestinal function remains unclear but many other phenomena, besides nutrient malabsorption, could underlie this observation.

### *Bcl-2* is dispensable for regeneration following radiation

Recently it was reported that *Lgr5*^+^ cells are required for intestinal regeneration following irradiation-induced damage^23^,^24^. To investigate if *Bcl-2* deficient *Lgr5*^+^ cells remain capable of epithelial regeneration in this setting, we crossed *Bcl-2*^fl/fl^ mice with *Lgr5*-Cre^ER^ mice to generate *Lgr5*-Cre^ER^.*Bcl-2*^fl/fl^ mice in which the *Bcl-2* gene can be deleted following tamoxifen injection. *Lgr5*-Cre^ER^ mice display a marked mosaicism for *Lgr5* promoter-driven EGFP-IRES-Cre^ER^ expression, which allowed us to directly compare *Lgr5*-GFP^+^.*Bcl-2*^−/−^ crypts and *Lgr5*-GFP^−^.*Bcl-2*^+/+^ crypts. Efficient inducible deletion of *Bcl-2* was observed after tamoxifen induction by immunohistochemistry and PCR analysis and >90% of *Lgr5*-GFP^+^ crypts displayed *Bcl-2* locus recombination and had lost BCL-2 expression (Supplementary Fig. 4a–c). Of note, *Bcl-2* loss in the ISC compartment does not alter the proportion of *Lgr5*-expressing cells indicating that *Bcl-2* is non-essential to the crypt base columnar ISC compartment in homeostasis (Supplementary Fig. 4d). In addition, the *Lgr5*-Cre^ER^.*Bcl-2*^fl/fl^ model permitted us to confirm that also epithelial specific loss of *Bcl-2* does not impact on intestinal morphology, the abundance of differentiated cell types (Supplementary Fig. 5a–g) or the number of proliferating and apoptotic cells (Supplementary Fig. 6a–d).

**Figure 3:**
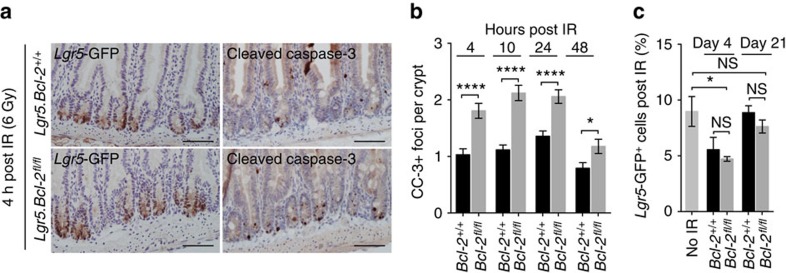
***Bcl-2*** **is dispensable for ISCs during regeneration.** (**a**) Panel of sections stained for cleaved caspase-3 (CC-3) in *Lgr5.Bcl-2*^+/+^ mice (top) and *Lgr5.Bcl-2*^fl/fl^ mice (bottom) 5 weeks after tamoxifen induction and 4 h after 6 Gy full-body irradiation. Scale bars, 100 μm. (**b**) Graph displaying the percentage of CC-3 foci per crypt for *Lgr5*.*Bcl-2*^+/+^ and *Lgr5*.*Bcl-2*^fl/fl^ mice at the indicated time points post 6 Gy irradiation. A minimum of 80 crypts was counted for each genotype and time point. Error bars represent the s.e.m., *P<0.05, ****P<0.0001, Student's *t*-test. (**c**) Graph showing the percentage of *Lgr5*-GFP^+^ cells in the small intestine for non-irradiated controls and post-irradiated mice at the indicated days (*n*=3 mice per condition, per time point). Analyses are performed by FACS. Error bars represent the s.e.m., NS, not significant, *P<0.05, Student's *t*-test.

In line with previous results assessing the role of *Bcl-2* in radiation resistance^25^,^26^, we observed that loss of *Bcl-2* results in increased apoptosis following a dose of 6 Gy irradiation as evidenced by cleaved caspase-3 staining at early time points (Fig. 3a,b). Although a decreased proportion of *Lgr5*-GFP^+^ cells can be detected early post-irradiation, 3 weeks after irradiation the number of *Lgr5*^+^ ISCs has restored to the untreated situation (Fig. 3c) and a similar trend can be observed for the number of *Lgr5*^+^ crypts (Supplementary Fig. 7a). Critically, we confirmed that the *Lgr5*-GFP^+^ cells that regenerated the intestinal tissue are *Bcl-2* deficient, ruling out the possibility that the regenerative potential resides in rare *Lgr5*^+^ cells that escaped *Bcl-2* loss (Supplementary Fig. 7b). Combined these results indicate that, following irradiation-induced DNA damage at this dosing regimen, *Bcl-2* protects against the occurrence of apoptosis in the crypt compartment but at the same time is dispensable for intestinal tissue regeneration.

### *Bcl-2* facilitates tumourigenesis in ISCs

We identified *Bcl-2* as a putative shared signal between ISCs and differentiated cells harbouring active NF-κB signalling, and speculate that this molecule might be involved in facilitating transformation in both cell types. To test this hypothesis we employed mice that allow for inducible inactivation of the tumour suppressor gene *Apc* (*Apc*^fl/fl^). *Apc* is a negative regulator of the Wnt pathway and loss of APC function results in intestinal hyperproliferation and adenoma formation^27^. Inactivating mutations in the homologous human *APC* gene, initiate a sizeable proportion of human CRCs^28^.

**Figure 4:**
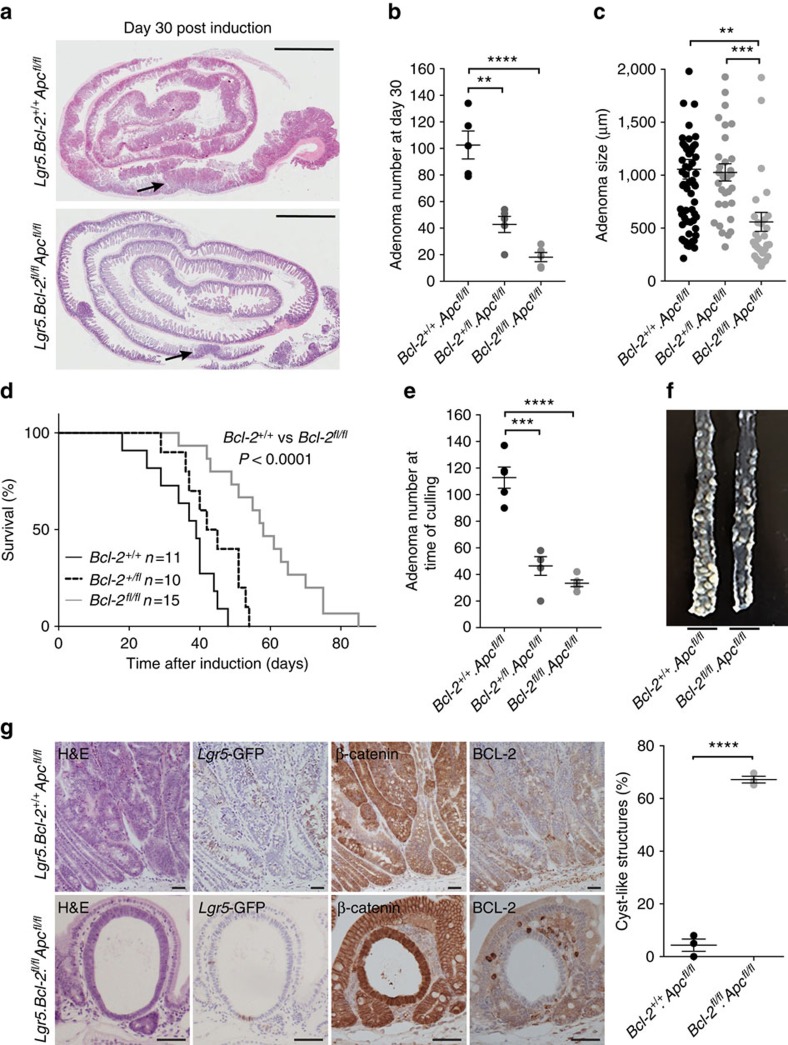
***Bcl-2***** loss impairs adenoma formation.** (**a**) Representative hematoxylin and eosin (H&E) stained sections of two small intestines of the *Lgr5*.*Bcl-2*^+/+^.*Apc*^fl/fl^ and *Lgr5*.*Bcl-2*^fl/fl^.*Apc*^fl/fl^ mice at day 30 post induction. Arrows indicate an adenomatous lesion. Scale bars, 2 mm. (**b**) Graph showing the number of adenomas at day 30 post induction for *Lgr5*.*Bcl-2*^+/+^.*Apc*^fl/fl^, *Lgr5*.*Bcl-2*^+/fl^.*Apc*^fl/fl^ and *Lgr5*.*Bcl-2*^fl/fl^.*Apc*^fl/fl^ mice. Each dot represents a mouse (*n*=5), error bars represent s.e.m. **P<0.01, ****P<0.0001, Student's *t*-test. (**c**) Graph showing the adenoma size at day 30 post induction in *Lgr5*.*Bcl-2*^+/+^.*Apc*^fl/fl^, *Lgr5*.*Bcl-2*^+/fl^.*Apc*^fl/fl^ and *Lgr5*.*Bcl-2*^fl/fl^.*Apc*^fl/fl^ mice. Each dot represents a measurement of the adenoma size (minimal *n*=32 per genotype), error bars represent the s.e.m. **P<0.01, ***P<0.001, Student's *t*-test. (**d**) Kaplan–Meier plot demonstrating the surviving fraction at the indicated time points post induction of *Lgr5*.*Bcl-2*^+/+^.*Apc*^fl/fl^, *Lgr5*.*Bcl-2*^+/fl^.*Apc*^fl/fl^ and *Lgr5*.*Bcl-2*^fl/fl^.*Apc*^fl/fl^ mice (minimal *n*=10 mice per genotype). *P* value by log-rank test. (**e**) Graph indicating the number of adenomas per mice at the time of culling in the survival analysis (*n*=4). Error bars represent the s.e.m., ***P<0.001, ****P<0.0001, Student's *t*-test. (**f**) Representative images of distal part of the small intestines of *Lgr5*.*Bcl-2*^+/+^.*Apc*^fl/fl^ and *Lgr5*.*Bcl-2*^fl/fl^.*Apc*^fl/fl^ mice, included in the survival analysis, at the time of termination. (**g**) Representative immunohistochemical panel of *Apc*-deficient adenomas (top panel) and cyst-like structures (bottom panel) in the small intestines of mice of the indicated genotypes 30 days after induction; H&E (left), *Lgr5*-GFP (middle-left), β-catenin (middle-right) and BCL-2 (right) staining. Scale bars, 50 μm. The graph displays the fraction of cyst-like structures observed in mice of the indicated genotypes at moment of termination. Each dot represents a mouse (*n*=3), error bars represent the s.e.m. ****P<0.0001, Student's *t*-test.

Here we crossed *Lgr5*-Cre^ER^.*Bcl-2*^fl/fl^ mice with *Apc*^fl/fl^ mice *(Lgr5*.*Bcl-2*^fl/fl^.*Apc*^fl/fl^) to determine the relevance of *Bcl-2* for intestinal tumour initiation. Critically, we confirmed all mice were highly congenic towards the C57BL/6 background by a single-nucleotide polymorphism (SNP) assay (Supplementary Fig. 8a,b). We observed that 30 days after tamoxifen administration, simultaneous inactivation of *Apc* and *Bcl-2* resulted in significantly impaired adenoma formation (Fig. 4a,b). Furthermore, even heterozygous loss of *Bcl-2* resulted in a threefold reduction in the number of adenomas (Fig. 4b). *Bcl-2* loss was associated with reduced adenoma sizes (Fig. 4c). In a separate experiment we studied the rate at which adenomas developed in the presence or absence of functional *Bcl-2*. We detected a significant delay in the onset of intestinal adenoma associated symptoms in mice that had impaired *Bcl-2* function within adenomatous clones (Fig. 4d). Mice that had to be killed in the *Lgr5*.*Bcl-2*^fl/fl^.*Apc*^fl/fl^ group had many fewer adenomas but suffered invariably from a few large tumours in the caecum that prompted killing of the mice (Fig. 4e,f). Importantly, we noticed a much higher number of cystic lesions in the intestines of *Lgr5*.*Bcl-2*^fl/fl^.*Apc*^fl/fl^ mice following induction (Fig. 4g). These lesions have previously been described as the consequence of inactivating *Apc* only in differentiated villus cells and contrast with the large, multiglandular adenomas that arise from ISCs^5^. Together these observations strongly suggest that *Bcl-2* is a critical gene required for stem cell initiated intestinal tumorigenesis.

### Inhibition of BCL-2 impairs intestinal tumorigenesis

**Figure 5:**
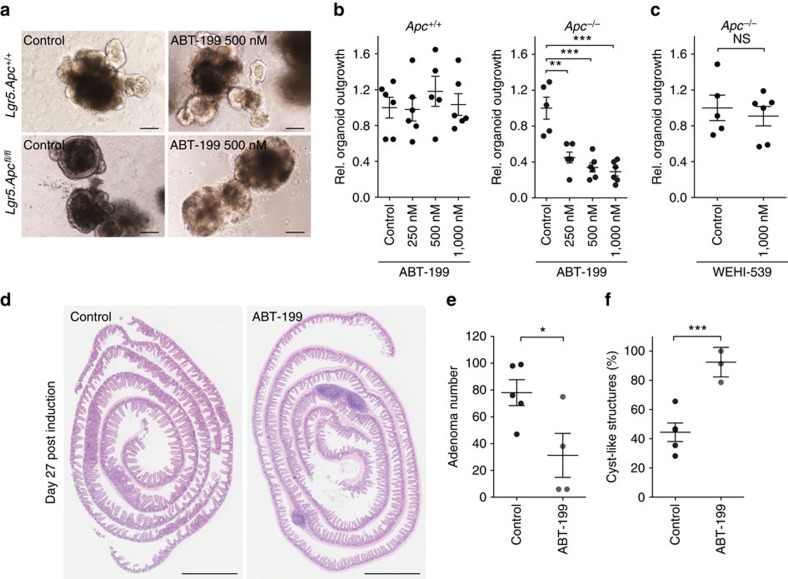
Pharmacological BCL-2 inhibition reduces tumor initiation. (**a**) Representative bright field images of untreated (left) and BCL-2 inhibitor (ABT-199) treated (right) organoid structures obtained from *Lgr5*.*Apc*^+/+^ (top) and *Lgr5*.*Apc*^fl/fl^ (bottom) mice 5 days after induction with tamoxifen. Scale bar, 50 μM. (**b**) Graphs depict the relative outgrowth of structures 7 days after passaging for the indicated genotype and ABT-199 dose at time of induction. Each dot represents a replicate (minimal *n*=5 per condition), error bars indicate the s.e.m. **P<0.01, ***P<0.001, Student's *t*-test. (**c**) Graph depicting the relative outgrowth of *Apc*^−/−^ structures 7 days after passaging treated with BCL-XL inhibitor (WEHI-539, 1,000 nM) at time of induction. Each dot represents a replicate (minimal *n*=5 per condition), error bars indicate the s.e.m. NS, not significant, Student's *t*-test. (**d**) Representative H&E images of sections of the small intestine of *Lgr5*.*Apc*^fl/fl^ mice, which were killed at day 27 post induction. The ABT-199 treated cohort received 100 mg kg^−1^ ABT-199 starting 2 days before induction for three consecutive days followed by administration every other day until day 27. Scale bars, 2 mm. (**e**) Graph displaying the number of adenomas at day 27 post induction. Each dot represents a mouse (minimal *n*=4 per condition), error bars represent the s.e.m. *P<0.05, Student's *t*-test. (**f**) Plot showing the quantification of the fraction of cyst-like structures in control and ABT-199 treated mice. Each dot represents a mouse (minimal *n*=3 per condition), error bars represent the s.e.m. ***P<0.001, Student's *t*-test.

Having identified *Bcl-2* as a critical facilitator of tumour formation, we wished to formally confirm that the consequences of impaired *Bcl-2* function are specific for *Apc* deficient cells. Therefore, we employed a clonogenic assay using intestinal organoid cultures derived from untreated *Lgr5-Cre*^*ER*^.*Apc*^fl/fl^ and *Lgr5-Cre*^*ER*^.*Apc*^+/+^ mice and, following tamoxifen induction *in vitro*, treated them with increasing doses of ABT-199, a selective BCL-2 inhibitor^29^. We observed that BCL-2 inhibition specifically reduced the outgrowth of *Apc*^−/−^ cells into spheroids, while leaving wild-type cells unaffected (Fig. 5a,b). These results were not recapitulated when using a specific inhibitor (WEHI-539) of another *Bcl-2* family member, BCL-XL, confirming that specifically BCL-2 inhibition is responsible for these observations (Fig. 5c). This is also in line with the observations following genetic *Bcl-2* ablation in our mouse studies. Next, we established that ABT-199 could prevent adenoma formation *in vivo*. Specifically, we found that oral application of the BCL-2 inhibitor, once every 2 days, in *Lgr5*-Cre^ER^.*Apc*^fl/fl^ mice for 27 days after tamoxifen administration resulted in a significant reduction in the average number of adenomas (Fig. 5d,e). We also corroborated our previous observation that transformation in a context of impaired BCL-2 function results in formation of indolent lesions, reminiscent of the cystic structures detected following *Apc* inactivation in differentiated cells (Fig. 5f).

### BCL-2 inhibition induces cell death in early adenomas

**Figure 6:**
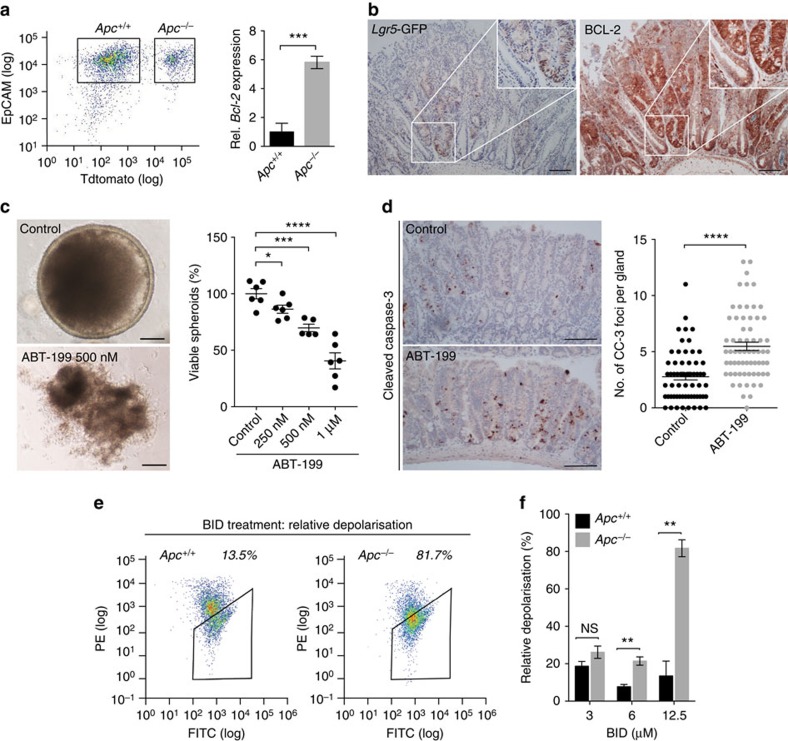
BCL-2 inhibition causes cells to undergo apoptosis in established adenomas. (**a**) Isolation of *Apc*^+/+^ and *Apc*^−/−^ cells at day 14 post low-level clone induction in *AhCre*^ER^.*Apc*^fl/fl^.*Lox-STOP-Lox*-*tdTomato*^+/−^ mice. The left panel represents the FACS sort analysis and the graph shows the relative *Bcl-2* expression of the *Apc*^+/+^ compared with the *Apc*^−/−^ cell population. Error bars represent s.d. (*n*=3 per condition), ***P<0.001, Student's *t*-test. (**b**) *Lgr5*-GFP and BCL-2 stained adenomas in a *Lgr5*.*Apc*^fl/fl^ mouse 5 weeks post induction demonstrating overexpression of *Bcl-2* in the *Lgr5*-GFP^+^ adenomatous glandular structures. Scale bars, 100 μm. (**c**) Representative images of *Apc*-deficient spheroids; upper image showing a viable spheroid of the control group and the lower image demonstrating a non-viable spheroid as observed after ABT-199 treatment. The graph displays the amount of viable spheroids counted at 72 h after the start of ABT-199 treatment for the indicated doses. Scale bars, 50 μM. Dots indicate replicates (minimal *n*=5 per condition), error bars represent the s.e.m. *P<0.05, ***P<0.001, ****P<0.0001, Student's *t*-test. (**d**) *Lgr5.Apc*^fl/fl^ mice with established adenomas 4 weeks following induction were treated with ABT-199 for 4 consecutive days. Cell death within these adenomas was compared with cell death within adenomas of untreated *Lgr5.Apc*^fl/fl^ mice by counting the amount of cleaved caspase-3 (CC-3) positive foci for each glandular structure. Each dot represents a gland (minimal *n*=62 glands per condition), error bars indicate the s.e.m. ****P<0.0001, Student's *t*-test. (**e**) FACS graphs indicating the sorting gates for *Ah*Cre^ER^.*Apc*^+/+^ and *Ah*Cre^ER^.*Apc*^−/−^ cells that have lost a JC-1 signal. (**f**) Bar graph showing the percentage of cells undergoing mitochondrial depolarization for the indicated concentrations of the BID peptide. The percentage of cells undergoing mitochondrial depolarization was measured with JC-1 staining and was calculated relative to the negative (DMSO) and positive (CCCP) control.

To determine the importance of *Bcl-2* in established adenomas we evaluated *Bcl-2* expression in adenomatous tissue. First, we found that *Bcl-2* messenger RNA is upregulated following *Apc*-loss by isolating tdTomato^+^/*Apc*^−/−^ cells from *Ah*Cre^ER^.*Apc*^fl/fl^.Lox-STOP-Lox-*tdTomato*^+/−^ mice 14 days after induction (Fig. 6a). Second, we performed immunohistochemical analysis of adenoma tissue that developed in induced *Lgr5*-Cre^ER^.*Apc*^fl/fl^ mice, and in a spontaneous model of intestinal tumourigenesis (*Apc*^Min^)^30^. This analysis revealed increased BCL-2 expression in transformed glands compared with normal tissue (Fig. 6b; Supplementary Fig. 9a). We observed that, similar to normal crypts, BCL-2 expression matches *Lgr5*-GFP expression suggesting a specific role of *Bcl-2* in stem-like cells in adenomas. A similar pattern of BCL-2 distribution was seen in human adenomas (Supplementary Fig. 9b). We speculated that BCL-2 inhibition might promote cell death in established adenomas. To test this, we treated *Apc*-deficient organoids with a BCL-2 inhibitor and found a significant reduction in the percentage of viable structures after 72 h (Fig. 6c; Supplementary Fig. 9c). We corroborated this *in vitro* observation by treating adenoma bearing *Lgr5*-Cre^ER^.*Apc*^fl/fl^ mice (3 weeks after induction) with ABT-199 for four consecutive days, which revealed efficient induction of cell death within these lesions (Fig. 6d). Together these results suggest that *Apc*^−/−^ cells are dependent on *Bcl-2* for survival and clonogenic expansion, both during tumour initiation as well as in established adenomatous tissue.

### *Apc* deficiency primes intestinal cells for apoptosis

To further investigate if *Apc* depletion causes cellular stress that leads to increased apoptotic priming, we adopted a previously described mitochondrial priming assay^31^. This assay assesses the readiness of cells to undergo apoptosis when treating cells with increasing concentrations of pro-apoptotic peptides. *Ah*Cre^ER^.*Apc*^fl/fl^ organoid cultures allowed us to determine the sensitivity of *Apc* depleted cells to undergo apoptosis. After induction, control and *Apc* deficient cells were treated with different concentrations of a synthetic BH3-interacting domain death agonist (BID) peptide, which is a pro-apoptotic protein that binds anti-apoptotic *Bcl-2* family members, including BCL-2, and causes a shift in the balance towards apoptosis induction. Next, the percentage of cells undergoing mitochondrial depolarization, a critical event in the apoptotic cascade, was measured by flow cytometry (Fig. 6e). Our results indicate that a significantly higher fraction of *Apc* deficient cells undergo BID-induced apoptosis compared with control cells at increasing BID-peptide concentrations (Fig. 6f). Thus, *Apc* inactivation causes cellular stress that primes intestinal epithelial cells for apoptosis. However, *Bcl-2* expression alleviates this effect and contributes to efficient transformation of ISCs.

### *Bcl-2* allows transformation of differentiated cells

**Figure 7:**
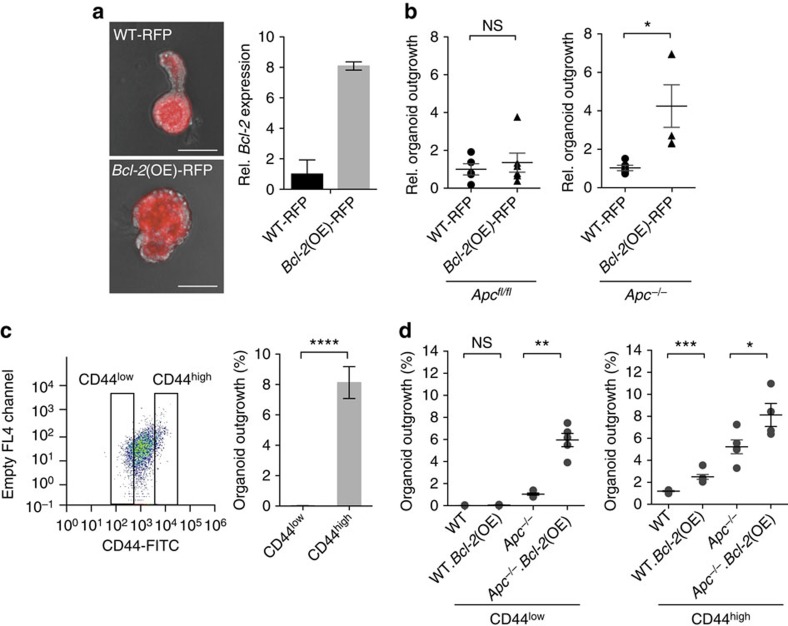
***Bcl-2*** **allows transformation of differentiated cells.** (**a**) Images displaying single-cell sorted RFP^+^ clones for the RFP-only transduced and *Bcl-2*(OE).RFP organoids (left panel). Bar graph showing gene expression analysis which indicates a eightfold increase in *Bcl-2* expression of the *Bcl-2*(OE) organoids compared with the wild type (WT) control. Error bars indicate s.e.m. (*n*=3 technical replicates per group). Scale bar, 100 μM. (**b**) Graphs displaying the relative outgrowth of WT-RFP and Bcl-2(OE)-RFP organoids after passaging in a control setting and with *Apc* inactivation. Each dot represents a replicate (minimal *n*=4 per condition), error bars indicate the s.e.m. NS, not significant, *P<0.05, Student's *t*-test. (**c**) FACS graph showing the sorting gates for CD44^low^ and CD44^high^ cells (left panel). Bar graph showing the clonogenic outgrowth of the CD44^high^ cells in comparison to the CD44^low^ cells (right panel). Error bars indicate the s.e.m. (minimal *n*=5 per condition), ****P<0.0001, Student's *t*-test. (**d**) Graph on the left displaying the outgrowth percentages of the CD44^low^ expressing cells and on the right of the CD44^high^ expressing cells; in both graphs the outgrowth percentage is shown for the WT, WT+*Bcl-2*(OE), *Apc*^−/−^ and *Apc*^−/−^+*Bcl-2*(OE) groups. For all conditions cells have been single-cell sorted followed by tamoxifen induction. Dots indicate replicates (minimal *n*=5 per condition), error bars represent the s.e.m. NS, not significant, *P<0.05, **P<0.01, ***P<0.001, Student's *t*-test.

Our results so far indicate that *Bcl-2* is an NF-κB target in intestinal epithelial cells and required for transformation of ISCs. Previously it was shown that differentiated epithelial cells with activated NF-κB signalling can effectively initiate adenoma formation^7^. Hence we hypothesized that expression of *Bcl-2* is sufficient for differentiated cells to withstand *Apc*-loss-induced apoptosis and generate clonogenic cells. To test this, we transduced *Ah*Cre^ER^.*Apc*^fl/fl^ organoid cultures with a *Bcl-2*-red fluorescent protein (RFP)-expressing vector (*Bcl-2*(OE)-RFP) or with an RFP-expressing control vector. We then generated single-cell clones and identified a line that showed physiological levels of *Bcl-2* (Fig. 7a). Overexpressing *Bcl-2* specifically enhanced the organoid forming capacity of cells that underwent *Apc*-loss, while had no effect on wild-type cells (Fig. 7b), suggesting that *Bcl-2* increases the population that can be successfully transformed. Subsequently, we stained the *Ah*Cre^ER^.*Apc*^fl/fl^ cultures for CD44 to separate the ISC and transit-amplifying cells (CD44^high^) from the more differentiated cells (CD44^low^; Fig. 7c). CD44^high^ cells efficiently generated new organoid cultures while CD44^low^ cells failed to do so, confirming the functional identity of both populations (Fig. 7c). Next, we tested the clonogenic outgrowth of the two populations (CD44^low^ and CD44^high^) both with and without *Bcl-2* overexpression following inactivation of *Apc* (Fig. 7d). In all cases *Bcl-2* overexpression increased the proportion of clonogenic cells likely reflecting that it prevents apoptosis caused by cellular stress associated with FACS sorting and single-cell plating. However, most importantly, *Bcl-2* overexpression greatly enhanced the capacity for clonogenic outgrowth of differentiated CD44^low^ cells that underwent *Apc* inactivation (Fig. 7d). This result suggests that *Bcl-2* overexpression can support transformation in differentiated cells not normally capable of clonogenic outgrowth.

## Discussion

**Figure 8:**
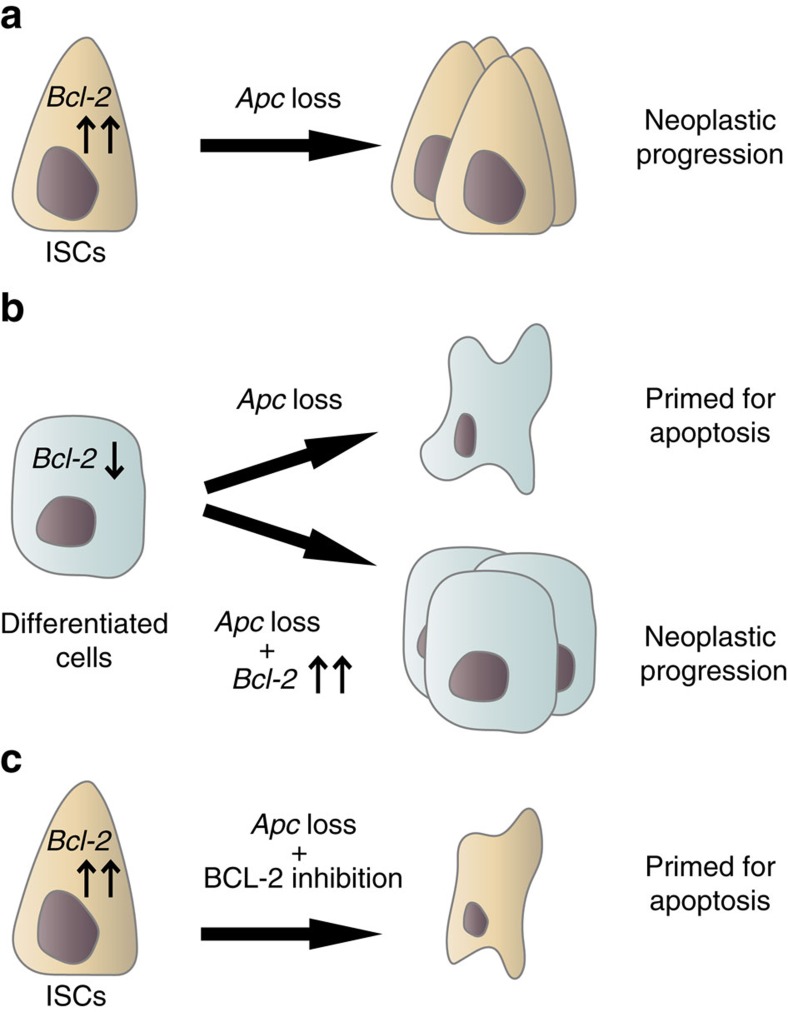
Model of the differential response to *Apc*-loss dependent on *Bcl-2* expression. (**a**,**b**) *Apc*-loss causes cellular stress which primes the *Bcl-2* deficient differentiated cells to undergo apoptosis whereas ISCs are rescued by high *Bcl-2* expression. As a result, only the ISC population is able to undergo malignant transformation upon *Apc* inactivation. However, when differentiated cells gain *Bcl-2* expression, these cells are also able to efficiently transform (**b**). This suggests that *Bcl-2* expression is an important prerequisite for intestinal epithelial cells to transform. (**c**) The survival of *Apc* depleted ISCs can be impaired by treatment with BCL-2 inhibitors and therefore we propose that BCL-2 could serve as an effective chemopreventive target for CRC development.

Previous studies on constitutive *Bcl-2* knockout mouse models demonstrated discrepancies regarding the effects of *Bcl-2* deficiency on the intestinal phenotype^20^,^21^,^22^. One study observed distorted villi throughout the small intestine and impaired proliferation^20^, whereas in two other models no intestinal abnormalities were detected^21^,^22^. Here we also show that *Bcl-2* is dispensable for intestinal homeostasis and no morphological impact of *Bcl-2* loss could be detected. In contrast, *Bcl-2* protects ISCs against irradiation-induced apoptosis, although *Bcl-2* deficient cells remain competent to contribute to intestinal regeneration. Our findings reveal that the NF-κB target gene *Bcl-2* is an important facilitator of oncogenic transformation within the intestine. A model summary of our findings is presented in Fig. 8. We demonstrate that the loss of *Bcl-2* reduces outgrowth of oncogenic *Apc*-deficient clones. Critically the effects of *Bcl-2* loss are specific for (pre)malignant lineages. Hence our results uncover a synthetic lethal interaction between *Bcl-2* and *Apc*^32^. Correspondingly, we have shown that *Apc*-loss is associated with an increased intracellular stress leading to apoptotic priming that is normally being evaded by BCL-2 function and by *Bcl-2* upregulation in *Apc*^−/−^ adenomatous tissue. This is in line with previous investigations on the role of anti-apoptotic *Bcl-2* family members as synthetic lethal partners in the development of haematological malignancies. Interestingly, there appears to be a clear lineage specificity regarding which *Bcl-2* family members are required for malignant transformation of specific cell types. For example, *Bcl-xL* is required for lymphoma development in a *myc*-driven mouse model while *Bcl-2* is dispensable in this setting^33^,^34^. In contrast, for thymic lymphoma development in a *p53*-deficient mouse model *Bcl-xL* is superfluous and *Mcl-1* is essential^35^. Similarly, our own results indicate that also the stage of disease is related to which family members are most essential in blocking apoptosis^36^. In contrast to the early disease studied here, in established human CRCs BCL-XL seems more critical^36^. It will be interesting to investigate at what exact stage, or with what genetic aberration this change in dependency corresponds.

Strikingly, our data revealed an increased formation of cyst-like structures on combined *Apc* and *Bcl-2* loss within the *Lgr5*^+^ ISCs at the expense of adenoma formation. Morphologically identical cystic structures are a consequence of *Apc*-loss within the differentiated cell compartment^5^. Furthermore, the formation of similar structures was reported on simultaneous *Lgr5*-Cre^ER^-mediated intestinal inactivation of *Apc* and *Rac1*, an upstream activator of NF-κB (ref. 14). We assume these lesions do, in principle, also occur in models in which *Apc*-loss is induced in the whole-epithelial population, but in these cases the cysts are usually overgrown by the adenomatous expansions that occur from transformation of the stem cell pool. This suggests that these structures do not reflect a gained property of *Bcl-2* deficient cells, rather they are more visible given the lack of efficient adenoma formation. More dedicated studies are required to strengthen this amenable concept.

Overall these observations lead us to speculate that *Bcl-2* expression might be the critical mediator allowing NF-κB-activated intestinal epithelial cells to efficiently transform. This remains to be formally demonstrated *in vivo*, although we have already shown that *Bcl-2* expression is sufficient to render differentiated cells in organoid cultures amendable to transformation as they give rise to clonogenic cells.

Because BCL-2 inhibition impedes the process of adenoma formation, pharmacological inhibition could play a role as preventive strategy against the development of colon carcinomas in populations that are at high risk for developing this disease. In particular patients who are diagnosed with a genetically inherited disorder, like familial adenomatous polyposis or hereditary non-polyposis CRC (known as Lynch syndrome), have an enhanced lifetime risk of developing CRC and would greatly benefit from effective chemoprevention^37^. The need for such interventions is emphasized by the fact that current chemoprevention regimes, for example, daily intake of non-steroidal anti-inflammatory drugs, have not proven to be sufficient in circumventing the need for other strategies, like two yearly colonoscopies and colectomy in early adulthood, to avert malignant disease^38^. It is encouraging to note that the BCL-2 inhibitors have already been used successfully for other neoplastic diseases and are continuously improving with respect to the reduced side-effects^29^,^39^.

## Methods

### Animal experiments

*Lgr5*-EGFP-IRES-Cre^ER^, PGK-Cre, *Ah*Cre^ER^, *Bcl-2*^*fl*/fl^, *Apc*^fl/fl^ and Lox-STOP-Lox-(LSL)-tdTomato mice all have been described previously^19^,^27^,^30^,^40^,^41^,^42^,^43^. All mice were bred and housed according to UK Home Office Guidelines. The genetic backgrounds of mice were assessed at the DartMouse Speed Congenic Core Facility at the Geisel School of Medicine at Dartmouth. DartMouse uses the Illumina, Inc. (San Diego, CA) GoldenGate Genotyping Assay to interrogate 1449 SNPs spread throughout the genome. The raw SNP data were analysed using DartMouse's SNaP-Map and Map-Synth software, allowing the determination for each mouse of the genetic background at each SNP location. This analysis confirmed that all mice involved in the survival analysis were highly congenic towards the C57BL/6 background (Fig. 4d, and Supplementary Fig. 8). Tamoxifen was dissolved in sunflower oil and administered via intraperitoneal (i.p.) injections. *Lgr5*-EGFP-IRES-Cre^ER^. *Bcl-2*^fl/fl^ mice were induced with in total 6 mg tamoxifen i.p. on three consecutive days (2 mg per day). *Lgr5*-EGFP-IRES-Cre^ER^.*Apc*^fl/fl^.*Bcl-2*^fl/fl^ mice were respectively induced with 4 and 2 mg tamoxifen i.p. for two consecutive days. *Ah*Cre^ER^.LSL-tdTomato^+/−^ mice are i.p. induced with 40 mg kg^−1^ β-naphthoflavone and 2 mg tamoxifen. Mice received 6 Gy total body irradiation (2.5 Gy per min). For the survival analysis mice were terminated when showing symptoms of anaemia in combination with weight loss and/or other signs of physical discomfort. At time of inclusion both male and female mice between the ages of 6 and 12 weeks were used. All animals were cared for at the Cancer Research UK Cambridge Institute Biological Resources Unit according to Home Office regulations.

### Human materials

All biological materials were obtained with informed consent from all subjects according to the Medical Research Involving Human Subjects Act (WMO) following approval of the ethical medical committee of the Academic Medical Center Amsterdam.

### Drug administration

Mice were treated with the ABT-199 compound (purchased from Active Biochemicals) or vehicle-only at a dose of 100 mg kg^−1^ daily for each protocol. ABT-199 was dissolved in 60% phosal 50PG, 30% polyethylene glycol 400 and 10% ethanol and administered by oral gavage. Treatment started 2 days before tamoxifen induction. Short-term experiments involved treatment for 4 days and the maximum treatment duration for long-term experiments was 27 days with ABT-199 administration on every other day.

### Immunohistochemistry

Dissected intestines are prepared as swiss rolls and fixed in a 10% formalin solution for 24 h before paraffin embedding. Sections (3 μm) are stained for haematoxylin/eosin and alcian blue. Immunohistochemistry was performed according to standard procedures. Antibodies were used as follows: anti-murine BCL-2 1:100 (BD Pharmingen, 554218), anti-human BCL-2 1:100 (DAKO, clone 124), anti-cleaved caspase-3 1:100 (R&D Systems, AF835), anti-GFP (Abcam, ab13970), anti-KI67 1:1000 (Dako), anti-Lysozyme 1:500 (Dako, A0099 M7249), anti-RELA/P65 1:100 (Santa Cruz, sc-372) and anti-Villin 1:100 (Santa Cruz, sc-7672). Cystic lesions were defined as single, distended glandular structures of which the luminal side was covered with an epithelial monolayer.

### Crypt culture

Intestinal crypts were derived from the *Lgr5*-Cre^ER^ and *Lgr5*-Cre^ER^*.Apc*^fl/fl^ mice as indicated. Crypts were isolated and cultured from the small intestine as described previously^44^. In short, isolated intestines were opened longitudinally and villi were gently scraped off. The intestine was washed with PBS and cut into pieces of <0.5 × 0.5 cm. These intestinal tissue pieces were then incubated in 2 mM EDTA at 4 °C for 30 min. After removal of the EDTA solution, the crypts were collected in ice-cold PBS by vigorously shaking the intestinal pieces in a tube and passing them through a 70-μM cell strainer (BD Bioscience). Isolated crypts were seeded in matrigel in a pre-heated 24-well flat-bottom plate. Advanced DMEM/F12 medium (Invitrogen) was used containing the following growth factors: mouse EGF 50 ng ml^−1^ (Tebu-bio), R-spondin (conditioned medium) and Noggin (conditioned medium)^45^. R-spondin were produced by stably transfected 293T cells with a plasmid encoding mRSPO1-Fc provided by dr Calvin Kuo at Stanford University, La Jolla, CA. To allow *in vitro* recombination, (Z)-4-hydroxytamoxifen (Sigma) was added to the medium with a final concentration of 100 nM. Organoids derived from the *Ah*Cre^ER^.*Apc*^fl/fl^ strain were induced with 5 μM (Z)-4-hydroxytamoxifen and 10 μg ml^−1^ β-naphthoflavone. NF-κB was inhibited in organoid cultures using JSH-23 (40 μM) obtained from Sigma-Aldrich (J4455). NF-κB was activated using TNFα (25 ng ml^−1^) obtained from eBioscience (14-8321-62). Organoid cultures were transduced with pHEFTIR (control) or pHEFTIR-BCL2 (BCL-2) lentiviral constructs. The constructs were generated as described by Colak *et al.*^36^. Following lentiviral transduction, single-cell derived WT-RFP and *Bcl-2*(OE).RFP organoid cultures were established for further experiments.

### Clonogenic assay

Organoid cultures from *Lgr5-*Cre^ER^.*Apc*^fl/fl^ and *Lgr5-*Cre^ER^.*Apc*^+/+^ mice were plated at a density of 150 crypt structures per well. After 3 days organoids were simultaneously induced with (Z)-4-hydroxytamoxifen (100 nM) and treated with ABT-199 or WEHI-539 (MedChem Express, HY-15607) for 72 h. To determine clonogenicity each well was passaged and organoid outgrowth was quantified after 7 days by microscope.

### Flow cytometry

The BD FACSAria III and BD Influx cell sorter were used for flow cytometry analysis and cell sorting. The epithelial fraction is identified with an Alexa 546 labeled EpCAM antibody (Biolegend Cat. no. 118212) at a concentration of 0.5 μg ml^−1^. CD44 low and high fractions were identified with anti-mouse CD44 labelled with fluorescein isothiocyanate (eBioscience). Dead cells were excluded with 4,6-diamidino-2-phenylindole or propidium iodide staining.

### Chromatin immunoprecipitation analysis (ChIP)

ChIP assay was performed with a p65 antibody 1:100 (Cell Signaling, #8242). The SimpleChIP Enzymatic Chromatin IP Kit with magnetic beads (Cell Signaling) was used according to the manufacturer's protocol. Briefly, TNFα treated (25 ng ml^−1^) and untreated organoids were fixed with 1% formaldehyde for 10 min at room temperature. Glycine was added to quench cross-linking. After washing with ice-cold PBS, cells were lysed and digested with 5 μl micrococcal nuclease (2,000 gel units per μl) per sample for 20 min at 37 °C. Additionally, cells were sonicated to break the nuclear membrane. Lysates were incubated with a p65 antibody and Normal Rabbit IgG overnight at 4 °C. Protein G magnetic beads were added and samples were incubated for 2 h at 4 °C. Protein G magnetic bead pellets were washed with low- and high-salt washes. Next the chromatin was eluted from the antibody/Protein G beads and DNA was eluted with DNA elution buffer. The forward primer 5′-ACACTTTATGTGTGGTTAGAAAGGG-3′ and reverse primer 5′-ACCTGGATCTTTTCTAACAGGATG-3′ were designed to identify the *Bcl-2* promoter region and used for PCR and quantitative real-time PCR analysis. PCR analysis was performed with Platinum *Taq* DNA Polymerase (Invitrogen) and Quantitative real-time PCR was performed with Sybr green (SensiFAST) according to manufacturer's protocol.

### Apoptotic priming assay

Mitochondrial priming was measured as described by Ryan *et al.*^31^ *Ah*Cre^ER^.*Apc*^fl/fl^ organoid cultures have been treated with 5 μM (Z)-4-hydroxytamoxifen 4 days before profiling to generate a high percentage of *Apc* deficient cells. At day four both control and *Apc*-deficient crypt cultures were collected and trypsinized. Cells were washed in T-EB (300 mM Trehalose, 10 mM HEPES-KOH pH 7.7, 80 mM KCl, 1 mM EGTA, 1 mM EDTA, 0.1% BSA and 5 mM succinate) and then resuspended in T-EB at a concentration of 500,000 cells per ml. The cell solution (in total 100 μl) was then mixed at a 1:1 ratio with T-EB containing 20 μg ml^−1^ digitonin, 20 μg ml^−1^ oligomycin and various concentrations of the BID peptide (EDIIRNIARHLAQVGDSMDR; Eurogentec, Maastricht, The Netherlands) and incubated at room temperature for 90 min. Next, JC-1 staining (1 μM) was performed for 30 min at room temperature and mitochondrial depolarization was measured on the FACS Canto (BD Biosciences).

### Polymerase chain reactions

Primers have been designed to identify recombination events of exon 2. The forward primer 5′-GAGCTGGATTTGCAGGCAGTTATTT-3′ and reverse primer 5′–TATGATTAAGGGCATTTTCCCACCA-3′ flank exon 2 of the *Bcl-2* locus. Genomic DNA was extracted from tissue or sorted cell populations and lysed with Cell Lysis Solution (5 PRIME) with 0.4 mg kg^−1^ proteinase K for 3 h at 50 °C. Protein precipitation solution (5 PRIME) was used according to manufacturer's instructions.

### RNA isolation and quantitative real-time PCR

RNA is extracted with the Qiagen RNeasy Plus Micro Kit (cat no. 74034). Synthesis of complementary DNA is performed with the Roche Transcriptor High Fidelity cDNA Synthesis kit (cat no. 05091284001). *Bcl-2* (cat no. 4331182, RefSeq NM_009741.4) and *Rpl19* (cat no. 4331182, RefSeq NM_000981.3) Taqman probes have been used for the Sybr green quantitative PCR with reverse transcription which was performed under standard conditions. The ΔΔCt method has been applied for calculations of gene expression.

### Analysis of gene expression and GSEA

Publicly available data sets were downloaded and normalized using robust multi-array average^46^. GSEA was performed as previously described using the GSEA desktop application (V2.0.14) from the Broad Institute (http://www.broadinstitute.org/gsea/index.jsp)^47^. The NF-kB target gene signature was derived from Compagno *et al.*^48^ and includes 120 genes of which we manually identified the murine homologues. For the drug targets the gene ontology ‘drug target' category was used. Identification of differentially expressed genes was performed using a *t*-test and a threshold of *P*<0.01 was used to determine significance.

## Additional information

**How to cite this article:** van der Heijden, M. *et al.* *Bcl-2* is a critical mediator of intestinal transformation. *Nat. Commun.* 7:10916 doi: 10.1038/ncomms10916 (2016).

## Supplementary Material

Supplementary Information Supplementary Figures 1-10
