# Supplementary material for: Bcl-2 is a critical mediator of intestinal transformation
Source: Nat Commun. 2016 Mar 9;7:10916. doi: 10.1038/ncomms10916 (PMC4786877; doi:10.1038/ncomms10916)
Supplement: Supplementary Information — Supplementary Figures 1-10 [file ncomms10916-s1.pdf]

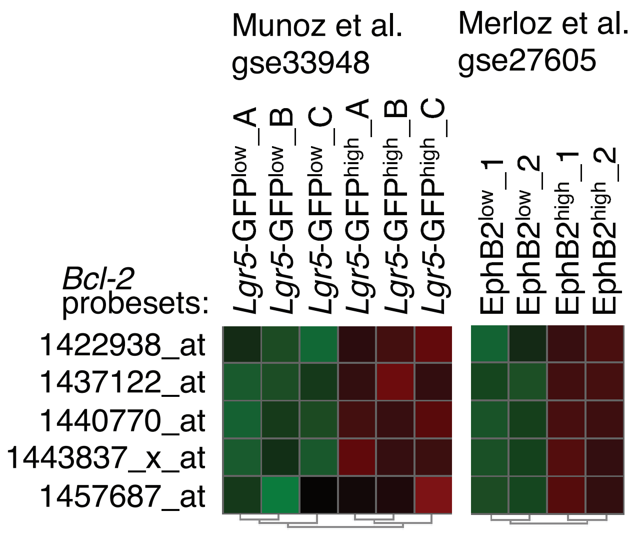

Supplementary Figure 1 | Gene expression analysis shows upregulation of *Bcl-2* in intestinal stem cells.

(**a**) Heatmap depicting upregulation of *Bcl-2* in the *Lgr5*-GFP^{high} vs. *Lgr5*-GFP^{low} and $EphB2^{high}$ vs. $EphB2^{low}$ cell population in the indicated publicly available datasets for the indicated probesets. Red is high expression, green is low expression.

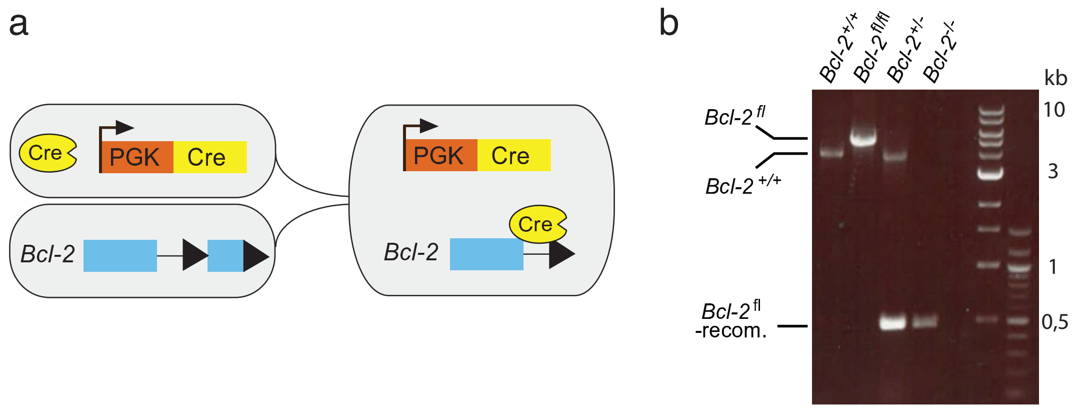

Supplementary Figure 2 | Validation of recombination of the *Bcl-2* locus in the *Bcl-2* knockout mouse model.

(**a**) Schematic overview of the *Bcl-2* knockout mouse model. (**b**) PCR analysis of *Bcl-2*$^{+/+}$ (WT), *Bcl-2*$^{fl/fl}$, *Bcl-2*$^{+/-}$ and *Bcl-2*$^{-/-}$ DNA for the indicated genotypes.

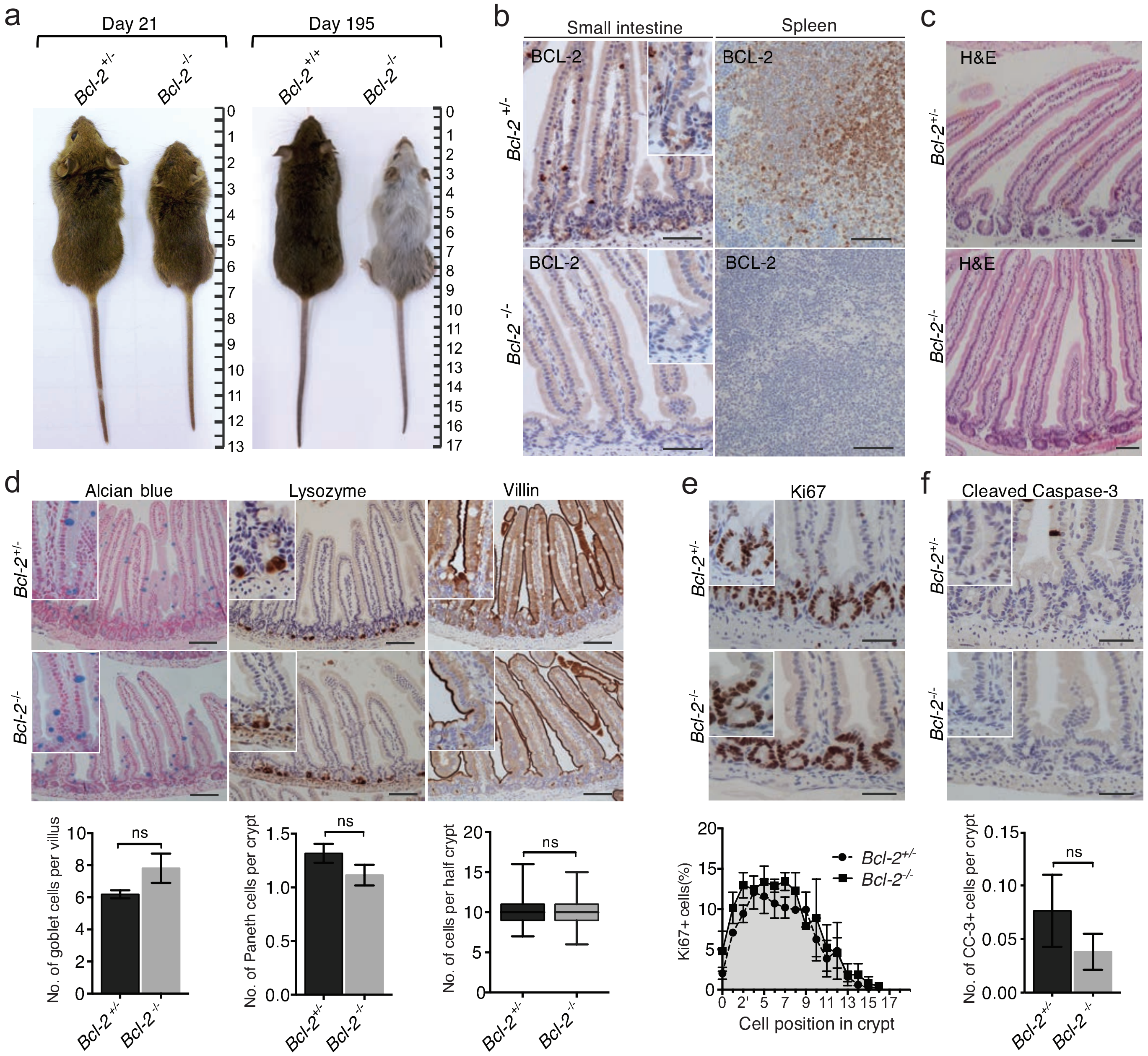

Supplementary Figure 3 | *Bcl-2* is dispensable for intestinal homeostasis.

(**a**) Images of *Bcl-2*$^{+/-}$ and *Bcl-2*$^{-/-}$ littermates at day 21 (left panel), and *Bcl-2*$^{+/+}$ and *Bcl-2*$^{-/-}$ littermates at day 195 after birth (right panel). Scale bar indicates cm. (**b**) BCL-2 stained sections of the murine small intestine (left) and spleen (right) of *Bcl-2*$^{+/-}$ and *Bcl-2*$^{-/-}$ littermates at day 21 after birth showing complete *Bcl-2* loss. Scale bars represent 100 µm. (**c**) H&E staining of small intestines of *Bcl-2*$^{+/-}$ and *Bcl-2*$^{-/-}$ littermates showing no morphological differences. Scale bar indicates 50 µm. (**d**) IHC panel representing the abundance of differentiated cell populations in the small intestine of *Bcl-2*$^{+/-}$ and *Bcl-2*$^{-/-}$ mice (top panel) and quantifications (bottom panel) of goblet and Paneth cells and the total amount of cells per half crypt. Scale bars represent 100 µm. (**e**) IHC depicting the abundance of proliferating cells within crypts. The upper panel shows Ki67 stainings for the indicated genotypes, the lower panel shows the distribution of Ki67 positive cells. Scale bars represent 50 µm. (**f**) Apoptotic cells are revealed by cleaved caspase-3 (CC-3) staining (upper panel) and the quantification (bottom panel). Scale bars represent 50 µm. (**d-f**) A minimum of 100 villi or crypts was counted for each genotype and time point. Error bars represent SEM, ns=not significant, Student's t-test.

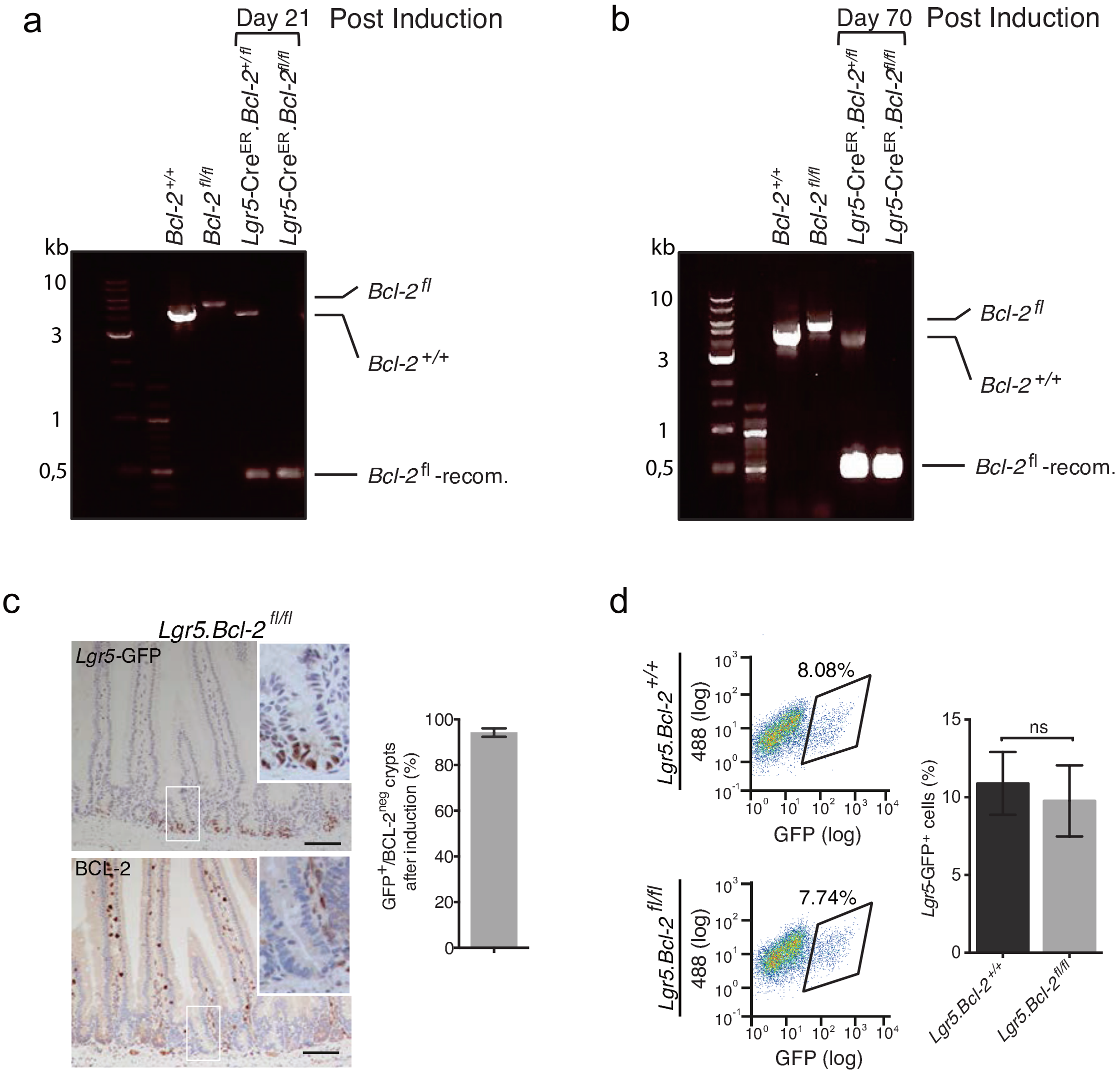

Supplementary Figure 4 | Validation of the *Lgr5.Bcl-2*$^{fl/fl}$ model.

(**a-b**) PCR validation of the recombined *Bcl-2*^{fl} locus post tamoxifen induction at day 21 and day 70. Control DNA is obtained from *Bcl-2*$^{+/+}$ and *Bcl-2*$^{fl/fl}$ mice. DNA of *Lgr5.Bcl-2*$^{+/fl}$ and *Lgr5.Bcl-2*$^{fl/fl}$ mice is obtained from FACS sorted *Lgr5*-GFP^{+} cells at the indicated time points post induction. (**c**) Validation of the recombination efficiency of the *Bcl-2* locus upon tamoxifen administration in the *Lgr5*-GFP^{+} crypt fractions by immunohistochemistry for *Lgr5*-GFP (top, left) and BCL-2 (bottom, left) in Lgr5.*Bcl-2*$^{fl/fl}$ mice. The corresponding quantifications of the percentage *Lgr5*-GFP^{+} crypts that lost *Bcl-2* expression (right panel). Error bar represents the SEM (n=3 mice). Scale bars indicate 100 µm. (**d**) The number of *Lgr5*-GFP^{+} cells in the small intestine was quantified by FACS analyses on day 4 after induction (left panel) with the corresponding quantification (right panel) showing no difference between the *Lgr5.Bcl-2*$^{+/+}$ and *Lgr5.Bcl-2*$^{fl/fl}$ mice. Error bars represent the SEM (n=3 mice), ns= not significant, Student's t-test.

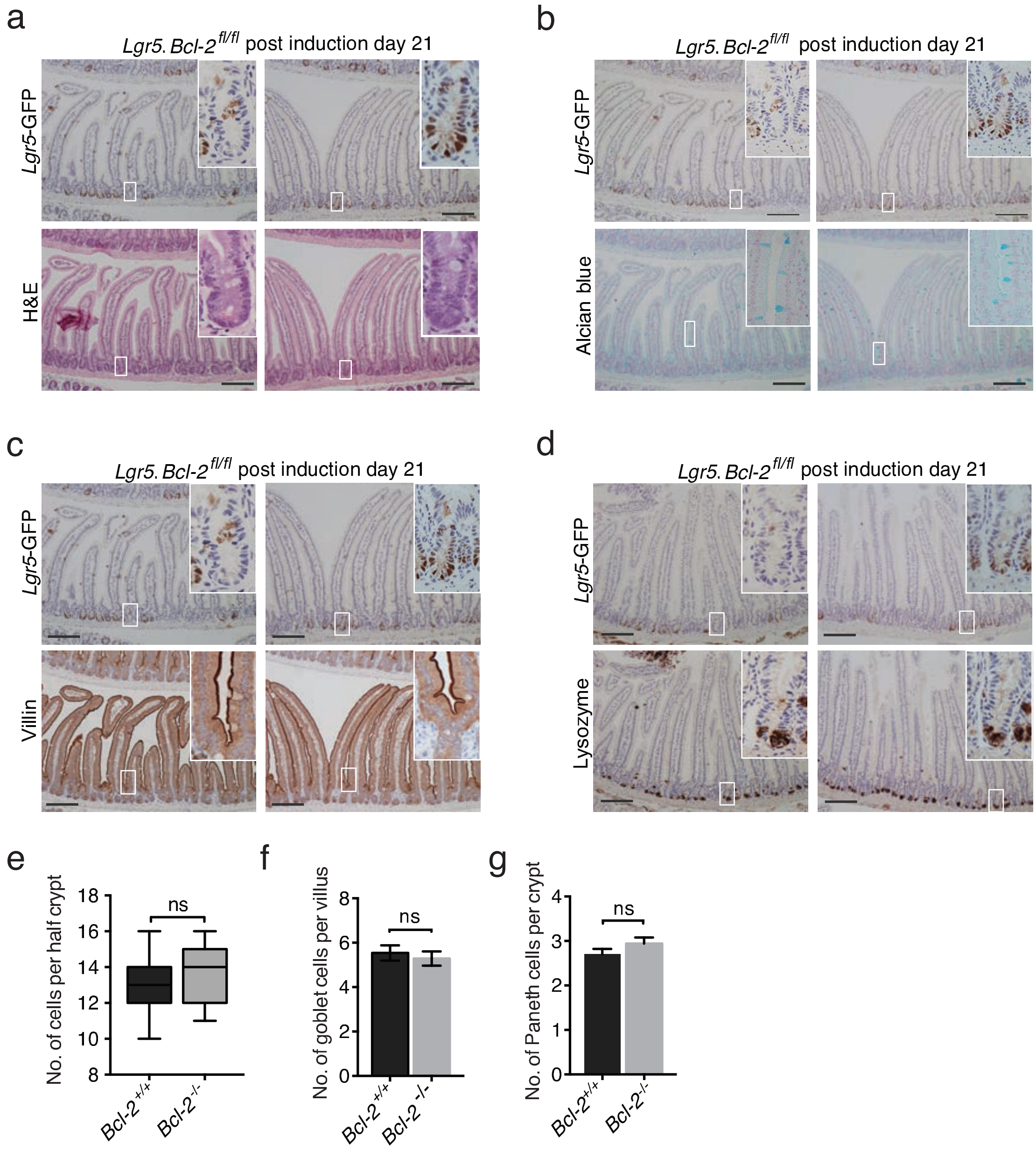

Supplementary Figure 5 | *Bcl-2* is dispensable for ISCs in homeostasis.

(**a**) Images of GFP and H&E stained sections of the indicated mice at day 21 after induction. Depicted are *Lgr5*-GFP$^-$ crypts, which are *Bcl-2*$^{+/+}$ and serve as an internal control for the *Lgr5*-GFP$^+$ crypts that are *Bcl-2*$^{-/-}$. (**b-d**) The images display GFP, alcian blue, Villin and Lysozyme and stained sections of intestines of the indicated mice that were collected 21 days after induction. (**e**) Graph showing the number of cells per half crypt indicating no difference in crypt size following *Bcl-2* loss. (minimal 80 crypts are scored per mouse and genotype, n=2 mice per genotype). Error bars represent the SEM, ns=not significant, Student's t-test. (**f-g**) Depicted are graphs showing the amount of goblet cells per villus (**f**) and Panet cells per crypt (**g**). A minimum of 70 crypts or villi was counted for each graph (n=1 mouse per genotype per time point). Error bars represent the SEM, ns=not significant, Student's t-test. Scale bars represent 200 µm.

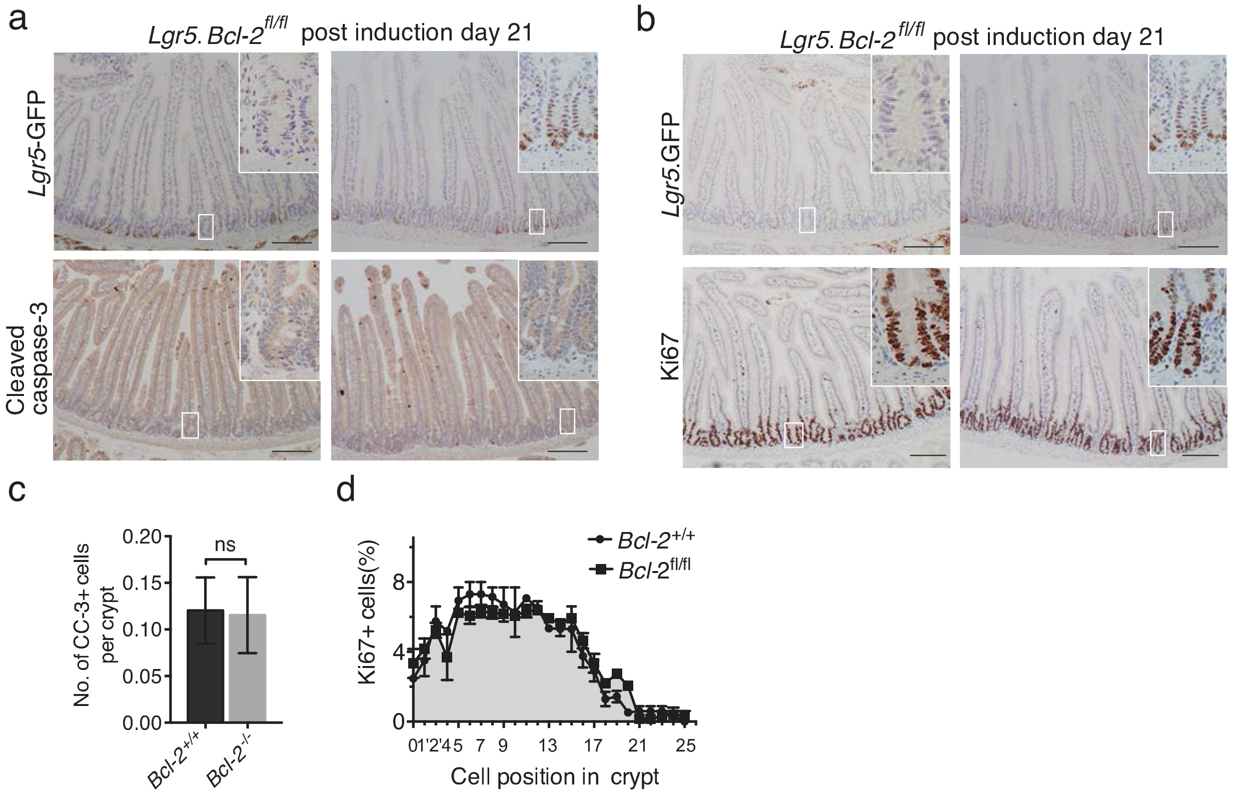

Supplementary Figure 6 | *Bcl-2* is dispensable for ISCs in homeostasis.

(**a**) Panel with GFP and cleaved caspase-3 (CC-3) stained sections of intestines collected at 21 days post induction for the indicated mouse strain. Depicted are *Lgr5*-GFP^{-} crypts, which are *Bcl-2*$^{+/+}$ and serve as an internal control for the *Lgr5*-GFP^{+} crypts that are *Bcl-2*$^{-/-}$. (**b**) Panel with GFP and Ki67 stained sections of intestines collected at 21 days post induction for the indicated mouse strain. (**c-d**) Graphs showing the number of CC-3 positive cells per crypt and the distribution of Ki67 positive cells within crypts. A minimum of 70 crypts was counted for each graph per genotype. Error bars represent the SEM, ns=not significant, Student's t-test. Scale bar represents 200 μm.

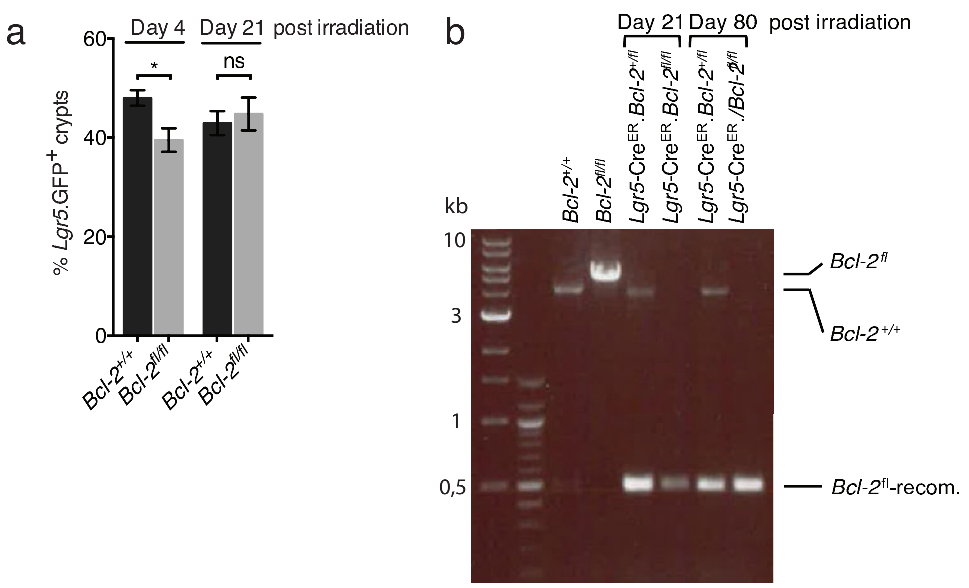

Supplementary Figure 7 | Irradiation does not affect the *Lgr5*.GFP^{+}.*Bcl-2*$^{-/-}$ population on long-term.
(**a**) Graph showing the percentage of *Lgr5*-GFP^{+} crypts after 6 Gy of irradiation at the indicated time points. Error bars represent the SEM (n=3 mice), ns=not significant, * $P<0.05$, Student's t-test. (**b**) PCR validating sustained presence of *Bcl-2*$^{-/-}$ cells post 6 Gy irradiation. Control DNA is obtained from *Bcl-2*$^{+/+}$ and *Bcl-2*$^{fl/fl}$ mice. DNA of *Lgr5.Bcl-2*$^{+/fl}$ and *Lgr5.Bcl-2*$^{fl/fl}$ mice is obtained from FACS sorted *Lgr5*-GFP^{+} cells at the indicated days after irradiation. Mice were administered tamoxifen two weeks prior to irradiation.

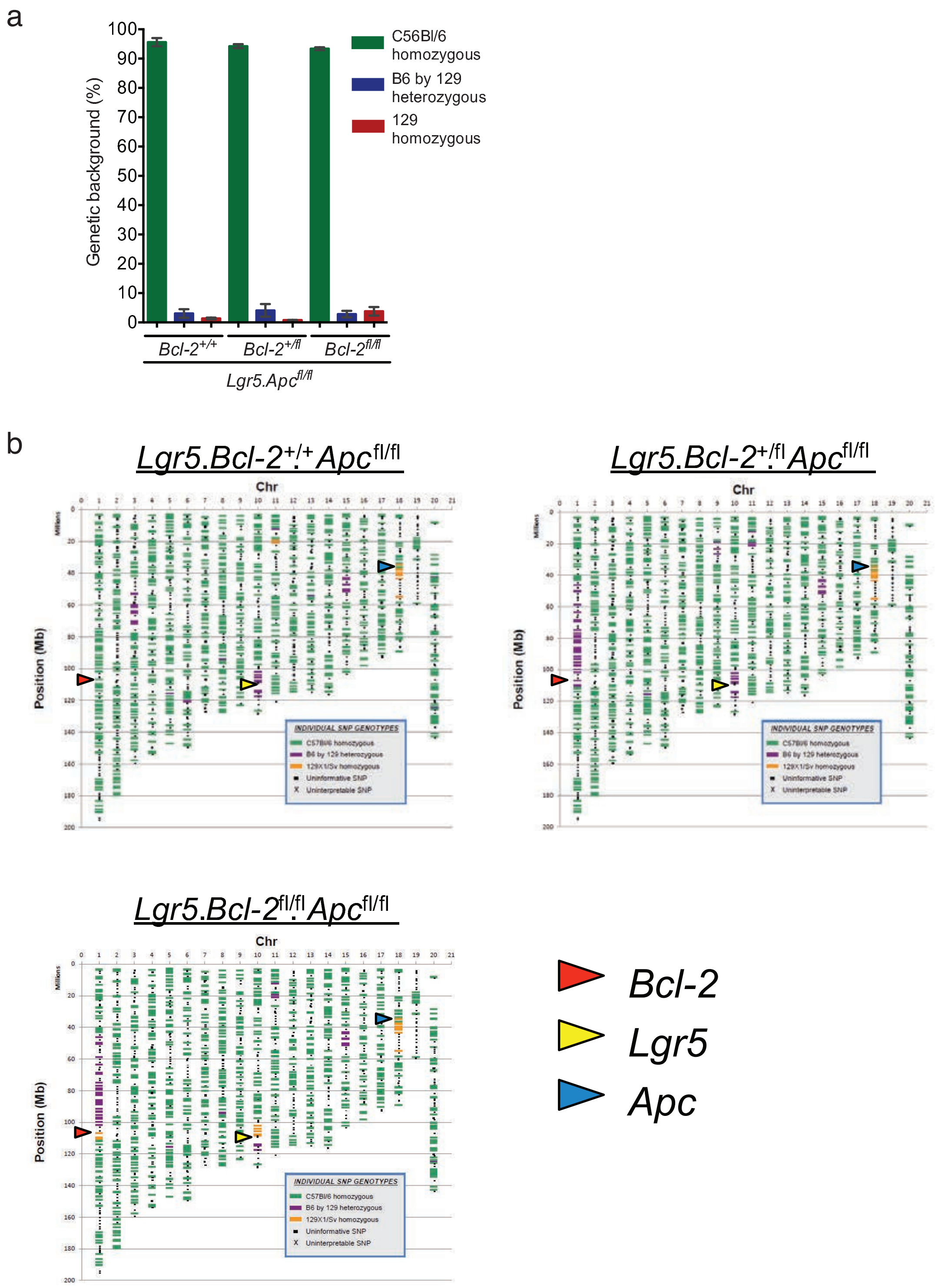

Supplementary Figure 8 | Genetic background screen of the *Lgr5.Bcl-2*$^{fl/fl}$.*Apc*$^{fl/fl}$ strain.

(**a**) Graph depicting percentages of the genomes of the indicated strains that are homo- or heterozygous for C56BL/6 and mixed background 129. Error bars represent the SEM (minimal n=3 mice per genotype). (**b**) Representative chromosome maps of the three different genotypes; *Lgr5.Bcl-2*$^{+/+}$.*Apc*$^{fl/fl}$, *Lgr5.Bcl-2*$^{+/fl}$.*Apc*$^{fl/fl}$ and *Lgr5.Bcl-2*$^{fl/fl}$.*Apc*$^{fl/fl}$. The genetically modified gene loci reside on the following positions; floxed *Bcl-2* gene ~106.5 Mbp on chromosome 1, *Lgr5* gene ~115.5 Mbp on chromosome 10 and floxed *Apc* gene ~34.2 Mbp on chromosome 18.

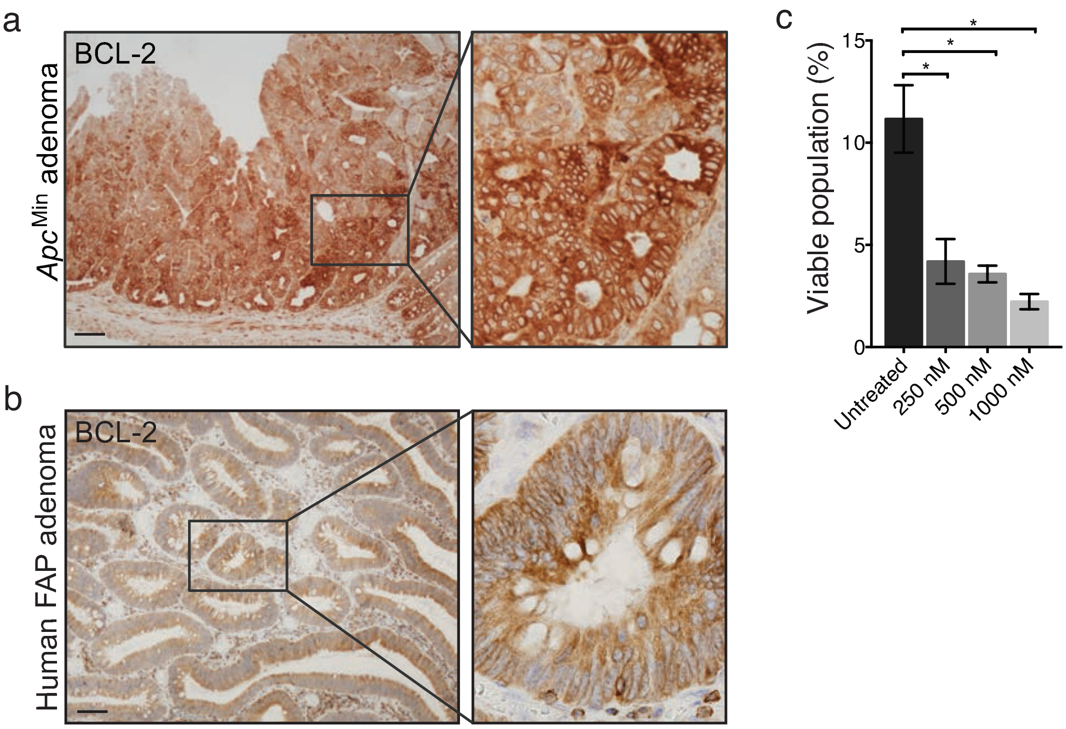

Supplementary Figure 9 | *Bcl-2* is highly expressed in adenomas and inhibition results in increased cell death in *Apc*$^{-/-}$ organoids.

(**a**) BCL-2 staining of an *Apc*^{Min} adenoma displays marked expression. Scale bar indicates 100 µm. (**b**). BCL-2 staining of human adenomatous tissue in an individual with familial adenomatous polyposis (FAP). Scale bar indicates 150 µm. (**c**) Graph indicating the percentage of viable cells of *Apc*$^{-/-}$ spheroids that are left untreated or have been treated for 72 hours with the indicated ABT-199 doses. The viable population for each condition is analysed by PI staining and flow cytometry. Error bars represent the SEM (n=3), * P<0.05, Student's t-test.

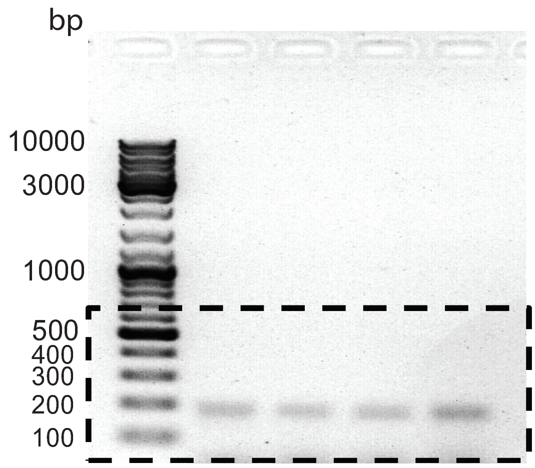

Supplementary Figure 10 | Full gel blot corresponding to figure 1f.
